# Frequency and Character of Extreme Aerosol Events in the Southwestern United States: A Case Study Analysis in Arizona

**DOI:** 10.3390/atmos7010001

**Published:** 2015-12-23

**Authors:** David H. Lopez, Michael R. Rabbani, Ewan Crosbie, Aishwarya Raman, Avelino F. Arellano, Armin Sorooshian

**Affiliations:** 1 Department of Chemical and Environmental Engineering, University of Arizona, Tucson, AZ 85721, USA; 2 National Aeronautics and Space Administration Langley Research Center, Chemistry and Dynamics Branch, Hampton, VA 23681, USA; 3 Oak Ridge Associated Universities, Oak Ridge, TN 37831, USA; 4 Department of Hydrology and Atmospheric Sciences, University of Arizona, Tucson, AZ 85721, USA

**Keywords:** aerosol, dust, IMPROVE, Asian dust, Arizona, air quality, extreme events

## Abstract

This study uses more than a decade’s worth of data across Arizona to characterize the spatiotemporal distribution, frequency, and source of extreme aerosol events, defined as when the concentration of a species on a particular day exceeds that of the average plus two standard deviations for that given month. Depending on which of eight sites studied, between 5% and 7% of the total days exhibited an extreme aerosol event due to either extreme levels of PM_10_, PM_2.5_, and/or fine soil. Grand Canyon exhibited the most extreme event days (120, *i.e.*, 7% of its total days). Fine soil is the pollutant type that most frequently impacted multiple sites at once at an extreme level. PM_10_, PM_2.5_, fine soil, non-Asian dust, and Elemental Carbon extreme events occurred most frequently in August. Nearly all Asian dust extreme events occurred between March and June. Extreme Elemental Carbon events have decreased as a function of time with statistical significance, while other pollutant categories did not show any significant change. Extreme events were most frequent for the various pollutant categories on either Wednesday or Thursday, but there was no statistically significant difference in the number of events on any particular day or on weekends *versus* weekdays.

## 1. Introduction

Severe aerosol pollution events pose a major threat to society due to significant reductions in visibility and air quality, in addition to adverse impacts on public health and daily operations. Fine particulate matter (PM_2.5_) is linked to various health impacts regardless of whether there is chronic or acute exposure, with effects ranging from lung cancer to cardiovascular disease [1,2]. Extreme pollution events are thought to be especially important with regard to annual acute mortality [3], in addition to leading to temporary shutdown of daily activities such as school and work in parts of the world [4]. Semi-arid and arid regions are particularly vulnerable to such events due to dust emissions, and this is especially dramatic during haboob events [5–7]. In recent decades, the Southwestern United States (Southwest) has experienced significant population growth, land use change, and is moving towards a more arid regime with higher temperatures, less precipitation, and lower soil moisture [8]. These changes promote increased dust emissions [9,10] and wildfires [11,12], with a rapidly growing population left vulnerable to the effects of the emissions. These issues coupled to the impact of dust and wildfire emissions on the hydrologic cycle and snowpack behavior at higher altitudes in the Southwest [9,13] warrants an examination of extreme aerosol events.

An ideal location to study extreme aerosol events is Arizona, which represents a state in the Southwest that is impacted by both dust and wildfires, in addition to having one of the fastest growing populations in the United States that is prone to the effects of poor air quality. The absolute population growth between 2000 and 2009, in Tucson and Phoenix, the two largest cities in Arizona, rank as the 33^rd^ and 4^th^ largest in the United States, respectively (U.S. Census Bureau, 2009). Sources of wind-blown dust impacting this and other Southwest states include naturally un-vegetated or anthropogenically disturbed soil surfaces, such as dry lakes (“playas”), dry washes, gravel pits, construction sites, oil and gas development sites, fields (after harvest), and long-range transport of Asian dust [14–20].

Aside from the ubiquity of dust in the Southwest, the greater Western United States is becoming increasingly vulnerable to the effects of wildfires owing to both a warmer climate and fire-control strategies over past decades resulting in conditions that promote larger and more frequent fires [11,21]. Depending on the fuel type and burning conditions, biomass burning leads to extensive emissions of various gaseous (e.g., nitrogen oxides (NO_x_), ozone (O_3_), carbon monoxide (CO), Volatile Organic Compounds (VOCs)) and particulate species (e.g., Elemental Carbon (EC), Organic Carbon (OC), inorganics), but also soil emissions due to lofting of soil in areas of turbulent mixing surrounding flames [22–24].

The goal of this study is to examine long-term data (2001—2014) from the EPA IMPROVE network across Arizona to characterize the frequency, spatial range, and origin of extreme aerosol events. The following questions are addressed: (i) what is the frequency of extreme aerosol events across Arizona and how many are due to EC-enriched air masses, Asian dust, non-Asian dust, or some other source? (ii) how frequently do these events occur at all or subsets of the study sites on the same day? and (iii) how are these events distributed between months of the year, days of the week, and inter-annually?

## 2. Experimental Methods

### 2.1. EPA IMPROVE

This study utilizes aerosol composition data from the Interagency Monitoring of Protected Visual Environments (IMPROVE) network [25]. IMPROVE aerosol monitoring stations are located primarily in National Parks and Wilderness Areas and collect ambient aerosol on filters over a period of 24 h every third day. Samples are analyzed for ions, metals, Organic Carbon (OC) and Elemental Carbon (EC). Among the elemental measurements, X-Ray Fluorescence (XRF) is used for Fe and heavier elements while Particle-Induced X-Ray Emission (PIXE) is used for elements Na to Mn. Fine soil concentrations reported in this study are calculated using the following equation [25]:

(1) $$\text{Fine Soil }(µg\cdot m^{-3})=2.2[Al]+2.49[Si]+1.63[Ca]+2.42[Fe]+1.94[Ti]$$

with regard to this equation, the components and their contributions were previously confirmed in comparisons of local re-suspended soils and ambient particles in the Western United States [25]. As this study is concerned with extreme concentrations of fine soil, it is expected that this equation can successfully capture all soil-rich air masses regardless of whether minor variations exist in the factors used in Equation (1). Species mass concentrations discussed in this study are from the fine fraction of aerosols (PM_2.5_). Sampling protocols and additional details are provided elsewhere [26].

**Figure 1:**
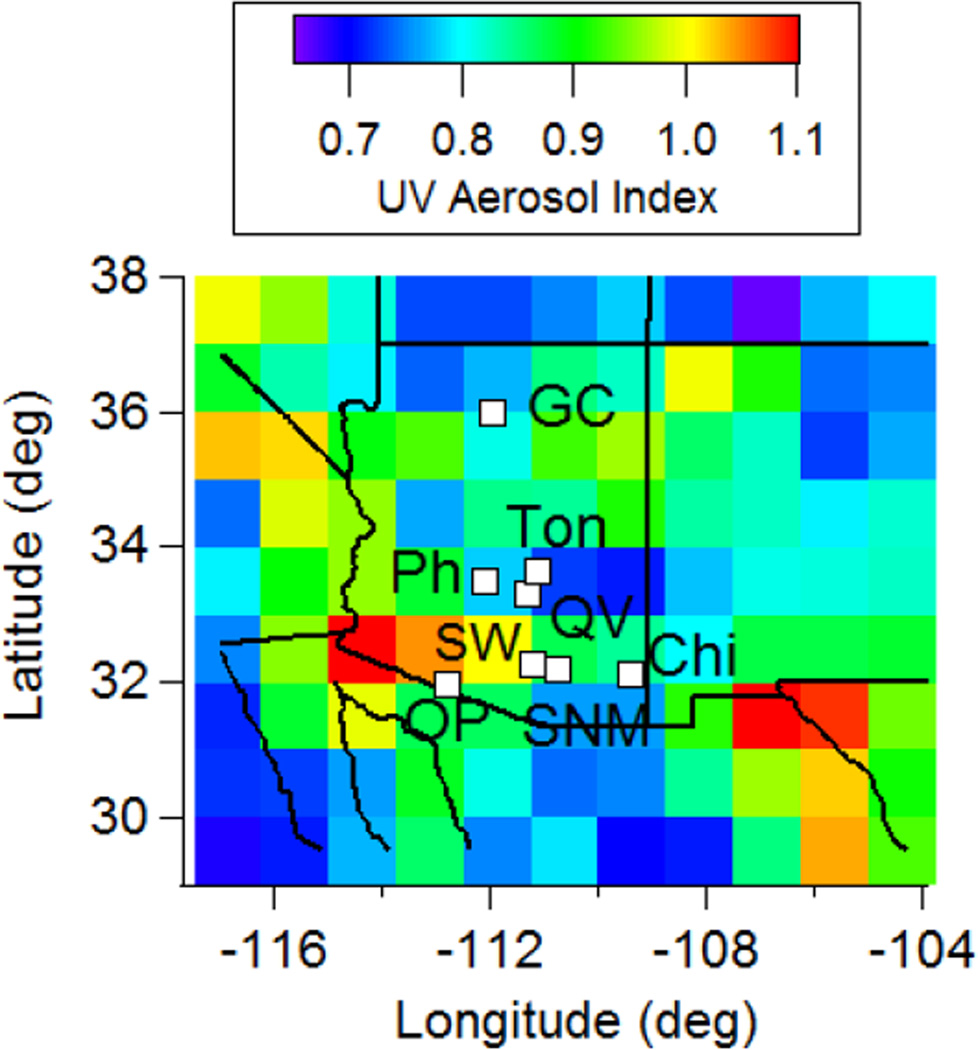
Spatial map of the eight EPA IMPROVE stations examined in Arizona overlaid on a four year average (2005–2008) of OMI ultraviolet aerosol index data, which includes influence from light-absorbing aerosol constituents, such as dust and smoke.

In this study, we use data from eight sites in Arizona (see map in Figure 1) and over time spans ranging from as early as data were possible starting in January 2001 until August 2014 (Table 1). Three of the eight sites are impacted more significantly by urban emissions owing to their closer proximity to populated cities; the Phoenix site is centrally located in the metropolitan area of the most populated city in Arizona, while Saguaro National Monument and Saguaro West are separated by ~50 km and are on the east and west sides, respectively, of Tucson, which is the second largest city. Chiricahua National Monument is a high-altitude site that is in a remote vegetated area with the nearest major urban area being Tucson (~150 km to the west). Nearby aerosol sources include the Willcox Playa and the Apache Power Plant, which are ~45 km to the west. This site is the closest to the Chihuahuan Desert. Tonto is ~90 km to the east/northeast of Phoenix and Queen Valley is ~60 km to the east of Phoenix, and thus these two sites are vulnerable to emissions transported from Phoenix. Organ Pipe is near the border of the United States and Mexico and is vulnerable to dust emissions and anthropogenic emissions from the nearby town of Sonoita, Mexico (~10,000 inhabitants). Grand Canyon is in a remote site in Northern Arizona and is removed from anthropogenic emissions.

### 2.2. NAAPS Aerosol Model

Simulation data providing information about long-range dust transport are obtained from the Navy Aerosol Analysis and Prediction system (NAAPS; http://www.nrlmry.navy.mil/aerosol_web/). NAAPS relies on global meteorological fields from the Navy Operational Global Atmospheric Prediction System (NOGAPS) [27,28] analyses and provides output at a spatial resolution of 1° × 1°, at six hour intervals, and with 24 vertical levels reaching 100 mb [29]. NAAPS has been used extensively to study intercontinental transport of dust to North America e.g., [18,29–32]. Sources of dust are defined in NAAPS using the USGS Land Cover Characteristics Database, which was created with Advanced Very High Resolution Radiometer (AVHRR) data. TOMS aerosol index data was used to further refine dust source regions. Dust emission occurs when the friction velocity exceeds a threshold value (value depending on land type) and when the surface moisture and snow depth are lower than a critical value (0.3 and 0.4 cm, respectively). The model operationally assimilates remotely-sensed aerosol optical depth (AOD) data from MODIS [33].

### 2.3. Satellite Data

Ultraviolet aerosol index (UV AI) data are obtained from the Ozone Monitoring Instrument (OMI). Data were obtained at a resolution 1° × 1.25° using a minimum threshold value of 0.5 [34]. The UV AI parameter serves as a proxy for absorbing aerosol particles [35], which are predominantly comprised of smoke and dust. Figure 1 shows a spatial map of a four-year average of OMI data to provide a backdrop of where light-absorbing aerosol particles (primarily dust in study region) are most abundant relative to where the eight IMPROVE stations are located.

### 2.4. Criteria for Events

In past work, criteria to define an extreme aerosol event have included the use of a cutoff threshold of a parameter value (*i.e.*, average ± *i* × standard deviation, with *i* starting at 1 and increasing; e.g., [36]) or when parameter values were below and above specific quantile values (e.g., 3). Here we take a similar approach to define an extreme event for specific aerosol parameters (e.g., PM_2.5_, PM_10_, and fine soil) as when the measured concentration on a given day at any of the eight sites exceeds the average concentration plus two times the standard deviation for the month in which the event occurred over the time range of data used for that particular site. This criterion leads to concentrations that exceed the 90^th^ percentile of mass concentrations in each category. The choice of this criterion reflects a balance between removing sensitivity to month-dependent factors and being sufficiently strict to isolate only a few cases that were the most polluted. The conclusions of this study, especially the number of extreme days in the various categories presented, are sensitive to the criteria definition. The numerical threshold criteria values (*i.e.*, average + 2 × standard deviation) for each site and month are shown in Table S1 (Supplementary Material).

Those events with extreme fine soil concentrations are referred to hereafter as extreme dust events. However, it is noted that extreme PM_10_ events that did not reach extreme fine soil levels could have also been due to dust that was concentrated in coarse aerosol (D_p_ ≥ 2.5 µm). Extreme fine soil events are further classified as having influence from Asia or not using output from the NAAPS model to validate long-range transport to the study region. The criteria for Asian dust was to observe a clear aerosol plume being advected from Asia to Arizona with multiple repeated NAAPS output plots as depicted in Figure S1 of the Supplement. It is cautioned that this classification scheme using NAAPS has limitations in that (i) the dust transport results are driven by a model rather than fully by observations, and (ii) the relative influence of Asian dust *versus* local sources is uncertain. Thus, although the term “Asian dust” is used subsequently, this is not meant to indicate that the fine soil measurement is fully due to long-range transport of dust from Asia. A suite of previous studies discussing source attribution of aerosol to long-range transport from Asia to North America have relied on NAAPS. For example, Cottle *et al.* [31] used NAAPS with HYSPLIT back-trajectories, and sunphotometer and lidar data to show that springtime dust plumes from Asia reached North America. Wu *et al.* [32], more recently, used NAAPS and remotely-sensed data from CALIPSO to study a trans-Pacific Asian dust event and its impact on the east coast of the United States. McKendry *et al.* [30] relied on the internal consistency between NAAPS and variety of other tools such as another global chemical model (GEOS-Chem) and surface and satellite observations to trace large dust plumes to their sources in areas, such as North Africa. The consistency between NAAPS and the other aforementioned resources provides confidence in the former for the purposes of source attribution of dust to Asia.

A category termed “High EC” is defined as when both PM_2.5_ and EC exhibit extreme levels. These events likely stem from anthropogenic sources and biomass burning events owing to the high levels of EC (as compared to other emission sources) and predominantly accumulation mode particles in wildfires [37]. Events that do not qualify as being extreme fine soil or High EC events are considered as “Other”.

It is cautioned that the number of extreme events reported between 2001 and 2014 represents an underestimate since data is used only up through August 2014 and only starts in January 2001 for three sites with the most delayed start time being for Organ Pipe in December 2002.

## 3. Results and Discussion

### 3.1. Frequency and Categorization of Events

Of the total number of days when data were available in the time ranges in Table 1 (*i.e.*, 1431–1664 depending on site), between 76 and 120 total days were characterized by some type of extreme event (*i.e.*, PM_10_, PM_2.5_, and/or fine soil) depending on the site (Table 2). This number of days of extreme events corresponds to between 5% and 7% of the total days examined. Grand Canyon exhibited the most extreme event days (120, *i.e.*, 7% of its total days), which is coincident with it being one of the most recognized tourism spots in the Southwest.

Relative to the total days with extreme PM_2.5_ levels, Grand Canyon exhibited the highest percentage in the High EC category (47% *versus* 13%–25% for other sites). Of the total number of days with extreme fine soil (54–69 days depending on the site), the number of these events being linked to Asian dust ranged from 19% to 29% (*i.e.*, 10–20 days). The total number of days with extreme events classified as Other (*i.e.*, not High EC or fine soil events) ranged from 17 to 30 days, which represents between 21% and 33% of the total extreme days depending on the site. The fact that the highest percentage of Other days were at Phoenix (31%) and Saguaro West (33%), the most urban-impacted sites among those studied, suggests that anthropogenic pollution, including anthropogenic dust, contributes to these events. Queen Valley also reached 31%, reflective of possible impact from transported pollution from the major nearby urban center Phoenix. Between 7 and 22 days in the Other category also registered extreme values of Coarse Mass (CM = PM_10_ − PM_2.5_), supporting the possibility of influence from locally generated dust.

To gain a sense of the spatial extent of pollution registering as extreme events, Table 3 shows how many sites experienced an extreme event for a specific pollutant category on the same day. Locally produced aerosol would not be expected to impact multiple sites at an extreme level as compared to a transported plume such as from Asia. Grand Canyon is farther removed from the other seven sites that are clustered closer in Southern Arizona, and, thus, Grand Canyon exhibits the highest number of days where an extreme event only impacted that site. Computed as a percentage of all extreme days registered for a particular pollutant type, Grand Canyon was the only site impacted out of 65%, 64%, 51%, 100%, and 84% of its extreme events for PM_10_, PM_2.5_, fine soil, High EC, and Other, respectively. The categories with the least number of extreme events impacting five or more sites were High EC (0 days for all sites) and Other (0–1 day depending on site). This result is thought to be due to locally generated pollution from either (i) some combination of biomass burning and anthropogenic activity (for High EC) or (ii) dust (for Other) that was not regional in nature. Fine soil events conversely impacted five or more sites on between 13 and 23 days, accounting for between 16% and 38% of all fine soil extreme events, depending on the site. Therefore, for the study region, fine soil is the pollutant type that most successfully impacts multiple sites at once at an extreme level.

The region-wide average for the PM_2.5_:PM_10_ ratio was 0.37, 0.35, and 0.23 for non-Asian dust, Asian dust, and Other-CM, respectively. Unexpectedly, the ratios for non-Asian dust events exceeded those for Asian dust events for half the sites (*i.e.*, Chiricahua, Phoenix, Saguaro National Monument, Saguaro West). Also of interest is that non-Asian dust event averages for PM_2.5_:PM_10_ were well above 0.35 at the two sites in Tucson, Arizona (Saguaro National Monument = 0.57 ± 0.36; Saguaro West = 0.46 ± 0.26). Aplausible explanation for these unexpected results is interference of background anthropogenic emissions at these urban-impacted sites in Tucson (Saguaro National Monument and SaguaroWest), in addition to Phoenix which exhibited an average ratio of 0.35. The same explanation can be applied to the Fe:Ca results, which do not show a clear reduction in value for Asian dust events as compared to the more locally-relevant pollution categories for Chiricahua, Saguaro National Monument, and Saguaro West. However, the region-wide average for Fe:Ca was lowest for Asian dust (0.88), followed by non-Asian dust (0.96), and Other-CM (1.03). The values were generally low and close to the threshold value applied by past work to classify dust as purely Asian dust. These results suggest that caution should be exercised with the use of such ratios to distinguish between dust sources owing to mixing between distant and local sources.

Due to the nature of Asian dust pollution being more geographically widespread than other forms of pollution, this category registered the highest frequency of its events impacting ≥5 sites. Depending on the site, 30%–57% of extreme Asian dust events (*i.e.*, 3–8 days) impacted ≥5 sites.

It is of interest to compare the Asian dust extreme event data to criteria used previously to distinguish Asian dust events in the study region, including mass concentration ratios of both Fe:Ca and PM_2.5_:PM_10_. Previous work showed that Fe:Ca ratios below 1 are considered to be 100% Asian dust and values above 2 are 100% local dust [38]. A threshold ratio value of 0.35 for PM_2.5_:PM_10_ has been applied in other work to remove contamination of non-local dust sources in the study region [39]. This ratio generally increases with dust plume age and, thus, values higher than 0.35 are assumed to be contaminated with sources such as transported Asian dust. Values between 0.15 and 0.26 are associated with soil dust emissions from human activities according to the EPA [39]. Table 4 examines statistics associated with the two aforementioned ratios for non-Asian dust, Asian dust, and also the subset of Other extreme events that also had extreme values of CM (PM_10_−PM_2.5_). The latter are presumed to be due to locally generated dust.

### 3.2. “Other” Events

The Other category was investigated in more detail to gain insight about the source of these extreme events (Table 5). Between 47% and 84% of the Other events exhibited extreme PM_10_ levels, which is suggestive of the presence of locally-generated coarse matter (*i.e.*, dust) since fine soil levels did not reach extreme levels. To gain confidence in this reasoning, the percent frequency of extreme CM days was calculated and is similar to the percent frequency of extreme PM_10_ days (*i.e.*, within two days) with the exception of Grand Canyon and Phoenix, which had nine and seven fewer extreme CM days as compared to PM_10_, respectively. Since the ratio of extreme CM:Other days ranges from 41% (Organ Pipe) to as high as 74% (Chiricahua), with the average among all sites being 56%, locally generated CM (*i.e.*, dust) accounted for a significant amount of the Other events.

PM_2.5_ levels reached extreme levels in 30%–76% of the Other extreme events, with Organ Pipe being the only site with a higher percentage for PM_2.5_ being extreme *versus* PM_10_. The PM_2.5_ constituents only reached extreme levels in an average of 10% (OC), 11% (K), 17% (nitrate), and 22% (sulfate) of the Other events. Among these four PM_2.5_ constituents, sulfate reached extreme levels in 48% and 35% of the Other events in Tonto and Organ Pipe, respectively, which were the highest values among all species and sites. This is likely due to anthropogenic emissions near those two sites such as from smelting [40–42]. Between 0% and 28% of Other events exhibited extreme levels of nitrate, OC, and potassium, which are all associated with wintertime pollution and fine soil emissions. These relatively low percentages for PM_2.5_ constituents are consistent with the majority of the Other events being due to CM.

### 3.3. Temporal Nature of Events

**Figure 2:**
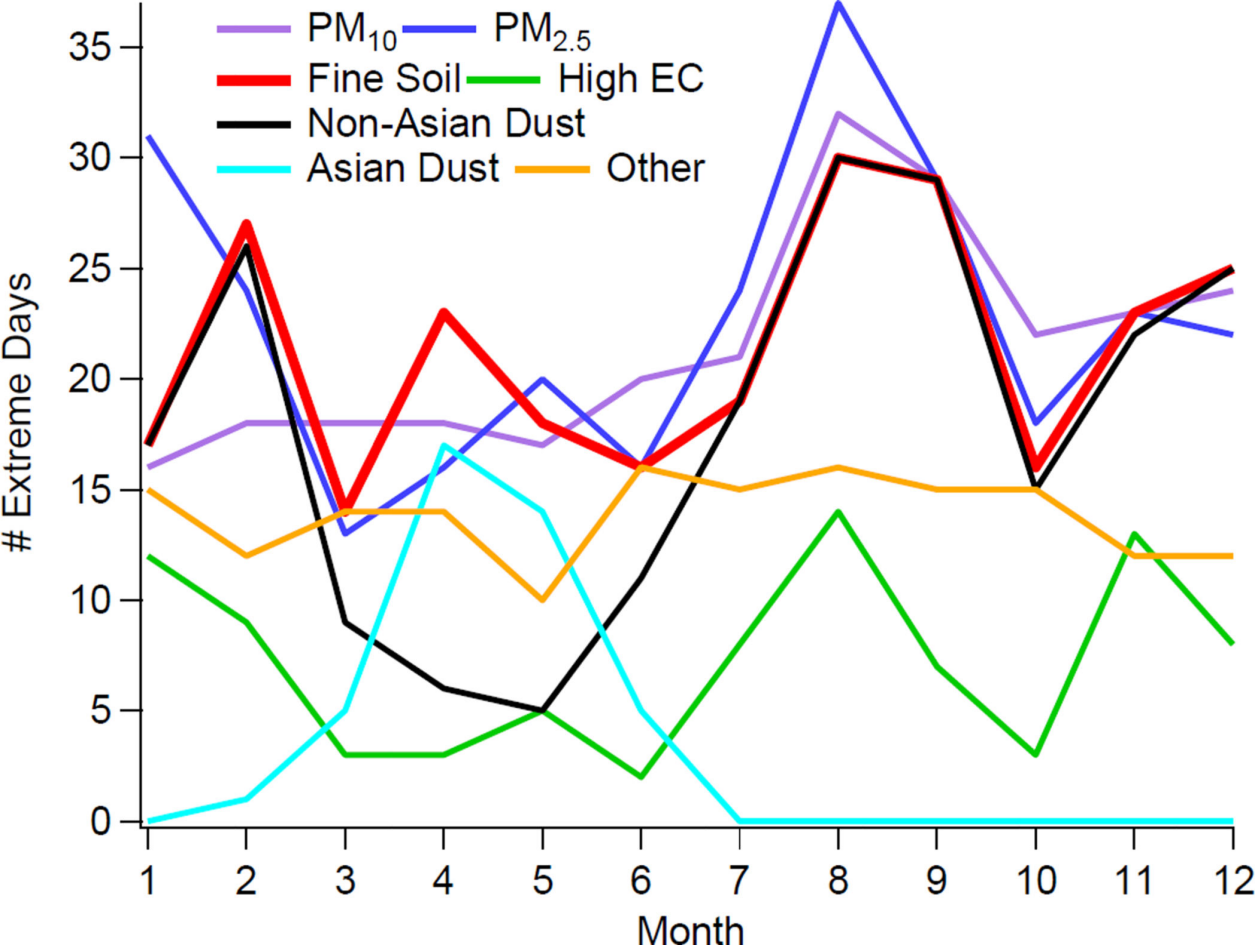
Monthly distribution of extreme events (cumulative for all years and sites) for different pollution categories.

Figure 2 displays the monthly distribution of cumulative (*i.e.*, summed for all years and sites) extreme events broken into the various pollutant categories. The month of August experienced the highest number of extreme events in the study region for PM_10_, PM_2.5_, fine soil, non-Asian dust, High EC, and Other (also had an equal peak in June). In contrast to all other pollutant categories, Asian dust events mainly occurred in the spring months of March–June (41 out of 42 days, *i.e.*, 98%) with only one event in February. The Other category exhibited a relatively constant amount in each month (12–16 days). Unlike PM_10_, PM_2.5_ exhibited a secondary mode in the winter month of January, driven mostly by High EC and Other events, suggestive of the importance of anthropogenic emissions and biomass burning, and secondary production of aerosol species that are favorably produced in wintertime conditions such as ammonium nitrate.

**Figure 3:**
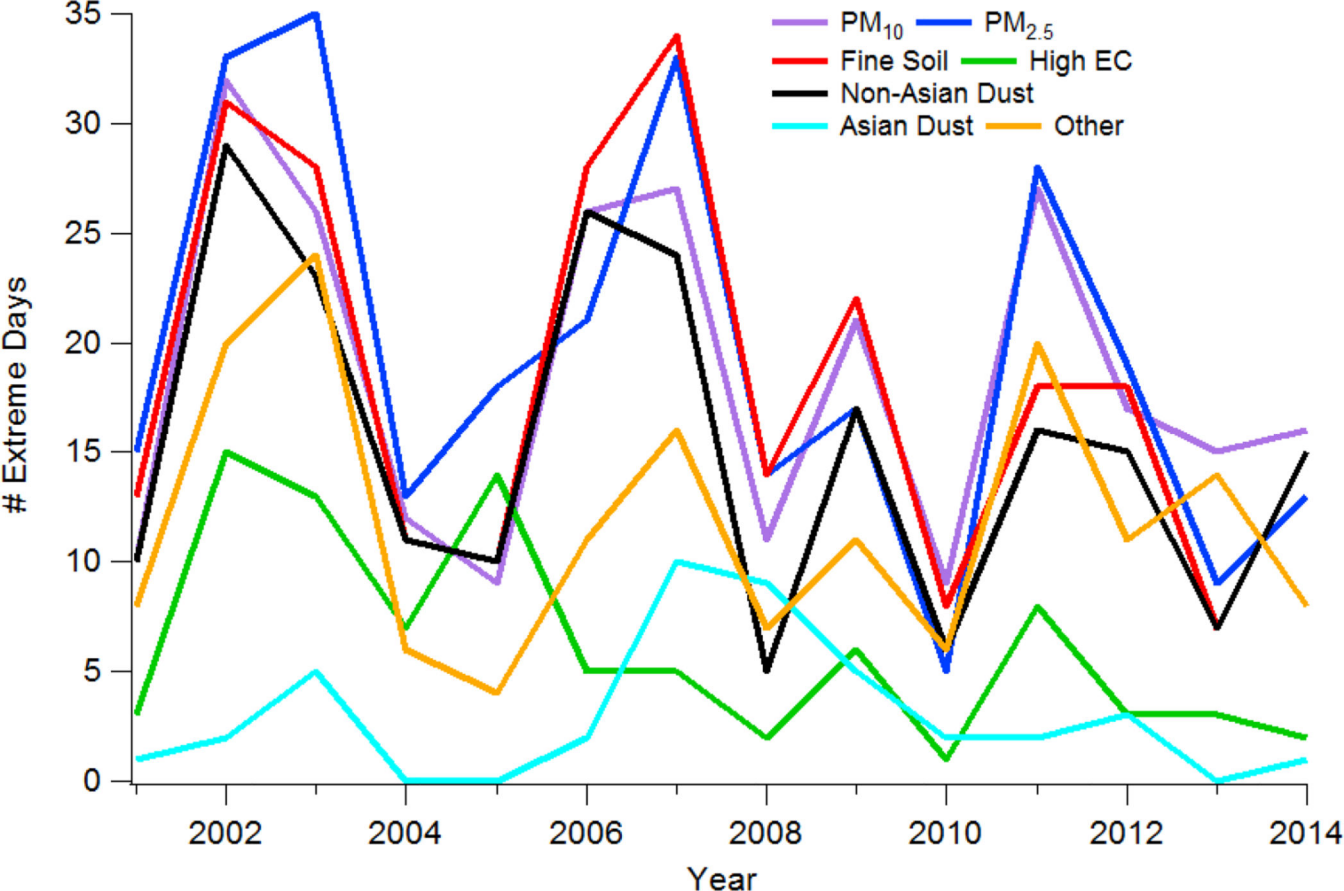
Time series of total extreme events as a function of year for different pollution categories. It is cautioned that the time range with full years of data at all eight sites is from 2003 to 2013 (refer to Table 1 for data time ranges for each site).

Figure 3 represents the interannual distribution of extreme events for different pollutant categories. It is cautioned that the time range with full years of data at all eight sites is from 2003 to 2013 (refer to Table 1 for data time ranges for each site). All categories exhibited the most events in either 2002 or 2003 with the exception of Asian dust which reached 10 events in 2007 and followed a distinctly different temporal pattern than all other categories due to its distant source. An interesting feature of Figure 3 is the cyclical pattern of there being a peak every few years for PM_10_, PM_2.5_, fine soil, non-Asian dust, and Other, specifically in the years 2002–2003, 2006–2007, 2009, and 2011–2012. It is unclear with the dataset as to what explains these recurring peaks, and future work is warranted, with a longer term record, to identify what an explanation could be for these features in the data.

A simple linear regression was used to obtain the best-fit line for each pollutant type in Figure 3 using data between 2003 and 2013 when data were available for all sites for full years. Most all slopes were negative except for the Other category, which was only barely positive. The slopes, reported in units of number of events per year, and p values (in parenthesis) are as follows: PM_10_ = −0.19 (0.80), PM_2.5_ = −1.13 (0.23), fine soil = −0.95 (0.29), High EC = −0.83 (0.03), non-Asian dust = −0.85 (0.25), Asian dust = −0.11 (0.76), Other = 0.03 (0.97). The only statistically significant trend at 95% confidence was for the High EC category. This is thought to be due to reduced anthropogenic emissions since other work for the study region examining 2005–2009 has shown that the fastest rate of decline in EC levels was in Phoenix [43], which is the most populated area. Another study analyzing IMPROVE data between 1990 and 2004 across the United States, including the Southwest, showed that there has been a ~25% reduction in EC attributed mostly to emissions controls, with the reduction being most dramatic in the winter as compared to summer [44].

The distribution of extreme events across days of the week is of interest for a few reasons. For example, EC rooted in anthropogenic emissions is thought to lead to higher concentrations around Thursday with minimum values on the weekend [44], and thus examining the frequency of High EC events as a function of the day of the week could help determine if the source of these events is anthropogenic in nature *versus* biomass burning. When normalized by total number of days on either the weekend (Saturday–Sunday) or weekday (Monday–Friday), High EC events occurred more frequently during weekdays (13.2 *versus* 10.5). All other categories exhibited more events during weekdays too. No air pollutant category exhibited a statistically significant difference in the number of events (normalized by number of either weekend or weekday days) on either the weekend or weekdays (or on any specific day) using a chi-square statistical test at the 95% confidence level. The day of the week with the most extreme events for the various pollutant categories was either Wednesday or Thursday.

## 4. Conclusions

The study examined long-term aerosol data for the Arizona region to describe the frequency and character of extreme aerosol events. The results are as follows in order of the questions raised at the end of Section 1:

- Between 5% and 7% of the total days (*i.e.*, 1431–1664 depending on site) examined at the various sites exhibited an extreme aerosol event due to either extreme levels of PM_10_, PM_2.5_, and/or fine soil. Grand Canyon exhibited the most extreme event days (120, *i.e.*, 7% of its total days), which is coincident with it being one of the most recognized tourism spots in the Southwest. Relative to the total number of extreme days, Grand Canyon exhibited the highest percentage in the High EC category (47% *versus* 13%–25% for other sites). “Other” events accounted for between 2% and 33% of the total extreme days, with most of these being associated with extreme PM_10_ levels (*i.e.*, locally-generated dust). Of the total number of days with extreme fine soil (54–69 days depending on the site), the number of these events being linked to Asian dust, based on NAAPS analysis, ranged from 19% to 29% (i.e., 10–20 days). The analysis highlighted the complexity of using NAAPS and various mass concentration ratios to distinguish between transported and local dust owing to likely mixing effects, especially in urban-impacted areas, such as Tucson and Phoenix.
- Fine soil is the pollutant type that most frequently impacted multiple sites simultaneously on the same day at an extreme level. Five or more sites reached extreme fine soil levels on the same day for 16%–38% of all possible fine soil extreme events depending on the site. Within the fine soil category, Asian dust events impacted five or more sites between 30% and 57% of the time when they occurred. The pollutant categories with the least number of extreme events impacting five or more sites on the same day were High EC (0 days for all sites) and Other (0–1 day depending on site) due to locally generated emissions that were not regional in nature. Grand Canyon exhibited the highest number of days where an extreme event only impacted that site since it is farther removed from the other seven sites that are clustered closer in Southern Arizona.
- Most pollutant categories (PM_10_, PM_2.5_, fine soil, non-Asian dust, High EC, Other) exhibited the highest number of extreme events in August. The Asian dust category was unique in its monthly pattern with its events occurring in the spring months of March–June (41 out of 42 days, *i.e.*, 98%) with only one event in February. Unlike the other pollutant categories, High EC was the only one to show a statistically significant change in frequency of occurrence between 2003 and 2013. While extreme events were most frequent for the various pollutant categories on either Wednesday or Thursday, there was no statistically significant difference in the number of events on any particular day or on weekend days *versus* weekdays.

## Supplementary Material

Supplement

## Figures and Tables

**Table 1:** Summary of IMPROVE sites and date ranges over which data are analyzed.

Site Name	Latitude (°)	Longitude (°)	Altitude (m)	Date Range
Chiricahua (Chi)	32.0994	−109.389	1554	January 2001–August 2014
Grand Canyon (GC)	35.9731	−111.9841	2267	January 2001–August 2014
Organ Pipe (OP)	31.9506	−112.8016	504	December 2002–August 2014
Phoenix (Ph)	33.5038	−112.096	342	April 2001–August 2014
Queen Valley (QV)	33.2939	−111.2858	661	April 2001–August 2014
Saguaro NM (SNM)	32.1746	−110.737	941	April 2001–August 2014
Saguaro West (SW)	32.2486	−111.2178	714	October 2001–August 2014
Tonto (Ton)	33.6548	−111.1068	775	January 2001–August 2014

**Table 2:** Statistics associated with the number of days with extreme events observed in the date range shown in Table 1 for each site.

Site Name	Total Days Data Available	Extreme Event Types							

		Total	PM_10_	PM_2.5_	Fine Soil	High EC	Non-Asian Dust	Asian Dust	Other
Chiricahua *	1664	89	61	56	54	14	41	10	23
Grand Canyon	1664	120	82	64	69	30	49	20	25
Organ Pipe	1431	76	45	55	54	7	41	13	17
Phoenix **	1628	98	54	58	63	9	50	10	30
Queen Valley ***	1628	98	65	60	60	9	43	15	30
Saguaro NM ***	1628	85	49	56	56	8	40	14	23
SaguaroWest ****	1563	91	56	55	55	10	44	10	30
Tonto ***	1664	103	67	65	67	12	47	18	25

(NAAPS global data were unavailable for the following dates in 2001 that are omitted from categorization into Asian and non-Asian dust: *16 October 2001, 26 August 2001, 9 November 2001; **16 October 2001, 31 October 2001, 21 November 2001; ***16 October 2001, 9 November 2001; ****9 November 2001). Values in the Total category represent days with any type of extreme event (*i.e.*, PM_10_, PM_2.5_, fine soil).

**Table 3:** Percentage breakdown (represented as fractions; *i.e.*, 0.1 = 10%) of the extreme events for different pollutant categories in terms of how many sites registered an extreme event for a particular pollutant on the same day. Each pollution category is separated into three columns representing extreme events occurring only at that site (1), 2–4 total sites, or 5–8 total sites.

Site Name	PM_10_			PM_2.5_			Fine Soil			High EC			Non-Asian Dust			Asian Dust			Other		

	1	2–4	5–8	1	2–4	5–8	1	2–4	5–8	1	2–4	5–8	1	2–4	5–8	1	2–4	5–8	1	2–4	5–8
Chiricahua	0.38	0.34	0.28	0.39	0.38	0.23	0.43	0.33	0.24	0.79	0.21	0.00	0.30	0.40	0.30	0.49	0.34	0.17	0.74	0.26	0.00
Grand Canyon	0.65	0.24	0.11	0.64	0.33	0.03	0.51	0.33	0.16	1.00	0.00	0.00	0.50	0.20	0.30	0.51	0.39	0.10	0.84	0.12	0.04
Organ Pipe	0.31	0.40	0.29	0.44	0.36	0.20	0.37	0.30	0.33	0.71	0.29	0.00	0.08	0.38	0.54	0.46	0.27	0.27	0.71	0.29	0.00
Phoenix	0.39	0.37	0.24	0.47	0.34	0.19	0.41	0.40	0.19	0.89	0.11	0.00	0.00	0.60	0.40	0.48	0.38	0.14	0.83	0.17	0.00
Queen Valley	0.25	0.45	0.31	0.27	0.47	0.27	0.22	0.40	0.38	0.56	0.44	0.00	0.00	0.47	0.53	0.30	0.40	0.30	0.57	0.40	0.03
Saguaro NM	0.20	0.45	0.35	0.27	0.50	0.23	0.27	0.36	0.38	0.75	0.25	0.00	0.21	0.21	0.57	0.30	0.43	0.28	0.74	0.22	0.04
Saguaro West	0.25	0.43	0.32	0.35	0.36	0.29	0.38	0.36	0.25	0.50	0.50	0.00	0.10	0.40	0.50	0.45	0.36	0.18	0.60	0.37	0.03
Tonto	0.18	0.51	0.31	0.20	0.57	0.23	0.13	0.55	0.31	0.75	0.25	0.00	0.11	0.44	0.44	0.15	0.62	0.23	0.60	0.36	0.04

**Table 4:** Average (±standard deviation) of two mass concentration ratios often applied to distinguish local dust from non-local dust (*i.e.*, Asian dust). Statistics are calculated for three extreme event categories: non-Asian dust, Asian dust, and both Other and CM simultaneously.

Site Name	PM_10_:PM_2.5_			Fe:Ca		
	
	Non-Asian Dust	Asian Dust	Other and CM	Non-Asian Dust	Asian Dust	Other and CM
Chiricahua	0.32 ± 0.17	0.30 ± 0.10	0.19 ± 0.08	0.82 ± 0.35	0.88 ± 0.24	0.95 ± 0.30
Grand Canyon	0.38 ± 0.11	0.43 ± 0.11	0.34 ± 0.06	0.83 ± 0.27	0.79 ± 0.21	0.92 ± 0.19
Organ Pipe	0.27 ± 0.10	0.39 ± 0.08	0.20 ± 0.09	0.98 ± 0.35	0.81 ± 0.16	0.96 ± 0.43
Phoenix	0.35 ± 0.16	0.30 ± 0.08	0.20 ± 0.07	1.27 ± 0.28	1.02 ± 0.25	1.22 ± 0.26
Queen Valley	0.27 ± 0.17	0.30 ± 0.10	0.18 ± 0.05	1.04 ± 0.35	0.84 ± 0.13	1.09 ± 0.40
Saguaro NM	0.57 ± 0.36	0.39 ± 0.13	0.27 ± 0.04	0.79 ± 0.28	0.80 ± 0.15	0.89 ± 0.24
SaguaroWest	0.46 ± 0.26	0.31 ± 0.12	0.21 ± 0.06	0.95 ± 0.48	1.04 ± 0.38	1.02 ± 0.44
Tonto	0.35 ± 0.19	0.38 ± 0.14	0.23 ± 0.08	1.00 ± 0.31	0.86 ± 0.15	1.16 ± 0.38

**Table 5:** Percentage frequency summary (represented as fractions; *i.e.*, 0.1 = 10%) of how many of the Other events at each site exhibited extreme levels of PM_10_, PM_2.5_, coarse mass (CM = PM_10_ − PM_2.5_) and individual PM_2.5_ constituents (potassium, organic carbon, nitrate, sulfate).

Species	Chiricahua (N = 23)	Grand Canyon (N = 25)	Organ Pipe (N = 17)	Phoenix (N = 30)	Queen Valley (N = 30)	Saguaro NM (N = 23)	Tonto (N = 25)
PM_10_	0.78	0.84	0.47	0.70	0.63	0.65	0.73
PM_2.5_	0.30	0.52	0.76	0.50	0.43	0.43	0.30
CM	0.74	0.48	0.41	0.47	0.63	0.57	0.73
K	0.09	0.24	0.12	0.17	0.10	0.09	0.03
OC	0.04	0.04	0.12	0.23	0.13	0.00	0.07
NO_3_^−^	0.13	0.20	0.18	0.17	0.10	0.17	0.10
SO_4_^2−^	0.17	0.08	0.35	0.07	0.20	0.26	0.13

**Table 6:** Day of week distribution of extreme events combining data from all eight sites over the entire time duration of the study. The number of extreme events on weekends and weekdays are shown with the values in parenthesis being the normalized values relative to the total number of weekend (2) and weekday (5) days. The day of the week with the most and least events are also shown with values in parenthesis being the actual number of occurrences on that particular day.

–	Total	PM_10_	PM_2.5_	Fine Soil	High EC	Non-Asian Dust	Asian Dust	Other
Weekend (Saturday–Sunday)	57 (28.5)	65 (32.5)	60 (30)	21 (10.5)	52 (26)	9 (4.5)	44 (22)	57 (28.5)
Weekday (Monday–Friday)	201 (40.2)	208 (41.6)	197 (39.4)	66 (13.2)	162 (32.4)	33 (6.6)	122 (24.4)	201 (40.2)
Day With Most Events	W (53)	Th (55)	W/Th (50/50)	Th (19)	Th (42)	W(11)	W(35)	W(53)
Day with Least Events	Tu (23)	Tu (25)	Tu (21)	M/F (11)	Tu (19)	Tu (1)	Tu (11)	Tu (23)
