# Supplementary material for: Frequency and Character of Extreme Aerosol Events in the Southwestern United States: A Case Study Analysis in Arizona
Source: Atmosphere (Basel). Author manuscript; Available in PMC 2016 Apr 13. (PMC4830501; doi:10.3390/atmos7010001)

Table S1. Summary of criteria concentrations for PM_10_, PM_2.5_, fine soil, and elemental carbon (EC) as a function of month for eight EPA IMPROVE sites in Arizona.

Figure S1. Case examples demonstrating how NAAPS was used as a tool to identify which extreme fine soil events qualified as Asian dust events. The examples below are for extreme fine soil events occurring on 15 May 2003, 12 April 2007, and 5 June 2008, which qualified as extreme events at four, six, and five sites, respectively.


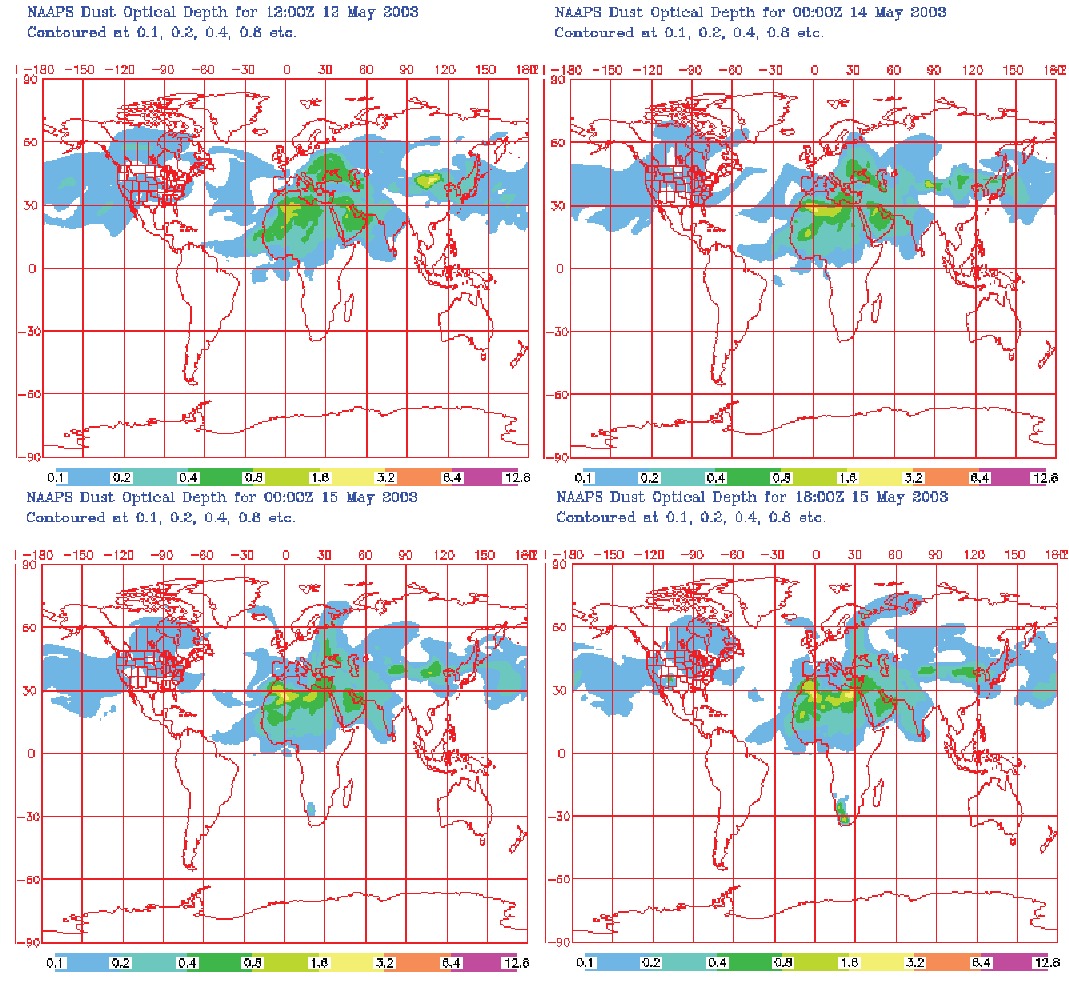


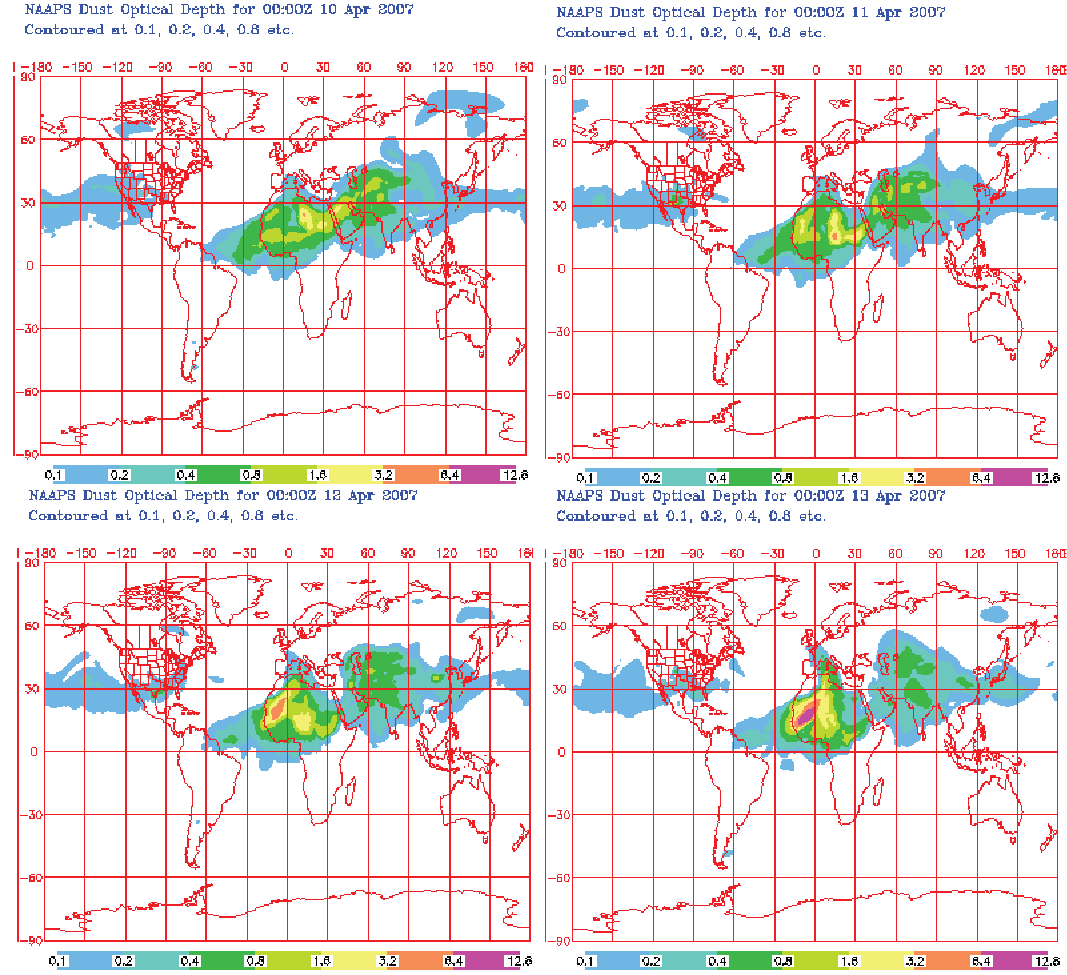


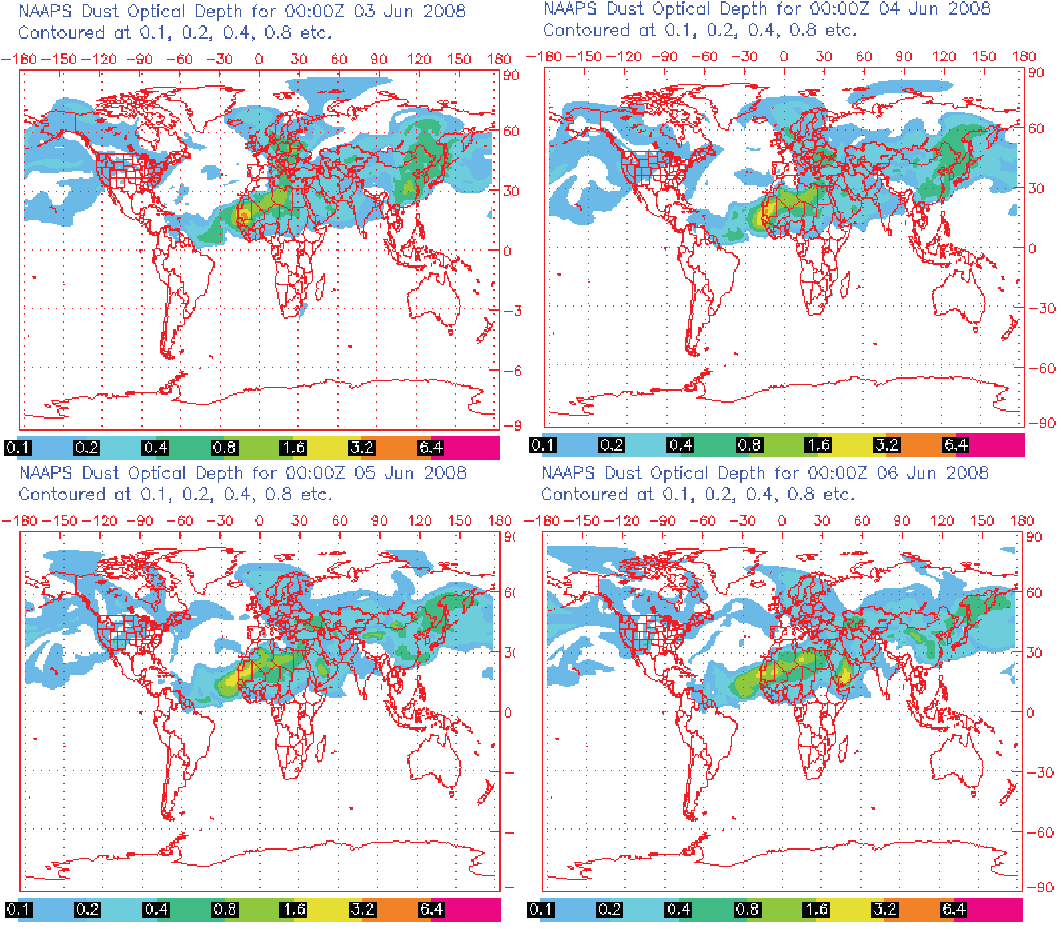

Supplement: Supplement [file NIHMS749773-supplement-Supplement.docx]
